# Sex Differences in Clustering Unhealthy Lifestyles Among Survivors of COVID-19: Latent Class Analysis

**DOI:** 10.2196/50189

**Published:** 2024-04-02

**Authors:** Lan T H Le, Thi Ngoc Anh Hoang, Tan T Nguyen, Tien D Dao, Binh N Do, Khue M Pham, Vinh H Vu, Linh V Pham, Lien T H Nguyen, Hoang C Nguyen, Tuan V Tran, Trung H Nguyen, Anh T Nguyen, Hoan V Nguyen, Phuoc B Nguyen, Hoai T T Nguyen, Thu T M Pham, Thuy T Le, Thao T P Nguyen, Cuong Q Tran, Ha-Linh Quach, Kien T Nguyen, Tuyen Van Duong

**Affiliations:** 1 Director Office Thai Nguyen National Hospital Thai Nguyen Vietnam; 2 Training and Direction of Healthcare Activity Center Thai Nguyen National Hospital Thai Nguyen Vietnam; 3 Biochemistry Department Thai Nguyen National Hospital Thai Nguyen Vietnam; 4 Faculty of Medicine Phenikaa University Hanoi Vietnam; 5 Department of Orthopedics Can Tho University of Medicine and Pharmacy Can Tho Vietnam; 6 Director Office Can Tho University of Medicine and Pharmacy Hospital Can Tho Vietnam; 7 Institute of Oncology and Nuclear Medicine Military Hospital 175 Ho Chi Minh City Vietnam; 8 Department of Infectious Diseases Vietnam Military Medical University Ha Noi Vietnam; 9 Department of Military Science Vietnam Military Medical University Ha Noi Vietnam; 10 Faculty of Public Health Hai Phong University of Medicine and Pharmacy Hai Phong Vietnam; 11 Infectious and Tropical Diseases Department Viet Tiep Hospital Hai Phong Vietnam; 12 Department of Pulmonary and Cardiovascular Diseases Hai Phong University of Medicine and Pharmacy Hospital Hai Phong Vietnam; 13 President Office Thai Nguyen University of Medicine and Pharmacy Thai Nguyen Vietnam; 14 Neurology Department Thai Nguyen University of Medicine and Pharmacy Thai Nguyen Vietnam; 15 Director Office Gang Thep Hospital Thai Nguyen Vietnam; 16 Director Office Hospital for Tropical Diseases Hai Duong Vietnam; 17 Department of Infectious Diseases Hai Phong University of Medicine and Pharmacy Hai Phong Vietnam; 18 Director Office Kien An Hospital Hai Phong Vietnam; 19 Training and Direction of Healthcare Activity Center Kien An Hospital Hai Phong Vietnam; 20 School of Public Health College of Public Health Taipei Medical University Taipei Taiwan; 21 President Office Da Nang University of Medical Technology and Pharmacy Da Nang Vietnam; 22 Faculty of Medical Laboratory Science Da Nang University of Medical Technology and Pharmacy Da Nang Vietnam; 23 Institute for Community Health Research University of Medicine and Pharmacy Hue University Hue Vietnam; 24 Faculty of Public Health Pham Ngoc Thach University of Medicine Ho Chi Minh City Vietnam; 25 Centre for Ageing Research & Education Duke-NUS Medical School National University of Singapore Singapore Singapore; 26 Department of Health Promotion Faculty of Social and Behavioral Sciences Hanoi University of Public Health Ha Noi Vietnam; 27 School of Nutrition and Health Sciences Taipei Medical University Taipei Taiwan; 28 International Master/Ph.D. Program in Medicine Taipei Medical University Taipei Taiwan

**Keywords:** sex difference, cluster, lifestyle behavior, COVID-19 recovery, latent class analysis, sex, unhealthy, lifestyle, adult, long COVID-19, infected, survivor, public health, intervention, promote, well-being, COVID-19, adults, mobile phone

## Abstract

**Background:**

The COVID-19 pandemic has underscored the significance of adopting healthy lifestyles to mitigate the risk of severe outcomes and long-term consequences.

**Objective:**

This study focuses on assessing the prevalence and clustering of 5 unhealthy lifestyle behaviors among Vietnamese adults after recovering from COVID-19, with a specific emphasis on sex differences.

**Methods:**

The cross-sectional data of 5890 survivors of COVID-19 in Vietnam were analyzed from December 2021 to October 2022. To examine the sex differences in 5 unhealthy lifestyle behaviors (smoking, drinking, unhealthy diet, physical inactivity, and sedentary behavior), the percentages were plotted along with their corresponding 95% CI for each behavior. Latent class analysis was used to identify 2 distinct classes of individuals based on the clustering of these behaviors: the “less unhealthy” group and the “more unhealthy” group. We examined the sociodemographic characteristics associated with each identified class and used logistic regression to investigate the factors related to the “more unhealthy” group.

**Results:**

The majority of individuals (male participants: 2432/2447, 99.4% and female participants: 3411/3443, 99.1%) exhibited at least 1 unhealthy behavior, with male participants being more susceptible to multiple unhealthy behaviors. The male-to-female ratio for having a single behavior was 1.003, but it escalated to 25 for individuals displaying all 5 behaviors. Male participants demonstrated a higher prevalence of combining alcohol intake with sedentary behavior (949/2447, 38.8%) or an unhealthy diet (861/2447, 35.2%), whereas female participants tended to exhibit physical inactivity combined with sedentary behavior (1305/3443, 37.9%) or an unhealthy diet (1260/3443, 36.6%). Married male participants had increased odds of falling into the “more unhealthy” group compared to their single counterparts (odds ratio [OR] 1.45, 95% CI 1.14-1.85), while female participants exhibited lower odds (OR 0.65, 95% CI 0.51-0.83). Female participants who are underweight showed a higher likelihood of belonging to the “more unhealthy” group (OR 1.11, 95% CI 0.89-1.39), but this was not observed among male participants (OR 0.6, 95% CI 0.41-0.89). In both sexes, older age, dependent employment, high education, and obesity were associated with higher odds of being in the “more unhealthy” group.

**Conclusions:**

The study identified notable sex differences in unhealthy lifestyle behaviors among survivors of COVID-19. Male survivors are more likely to engage in unhealthy behaviors compared to female survivors. These findings emphasize the importance of tailored public health interventions targeting sex-specific unhealthy behaviors. Specifically, addressing unhealthy habits is crucial for promoting post–COVID-19 health and well-being.

## Introduction

The COVID-19 pandemic has a significant impact on population health and causes widespread disruption globally [1]. Beyond the public health crisis, it has triggered substantial alternations in people’s lifestyles, including poorer nutrition intake, sedentary lifestyle due to prolonged lockdown, sleep disturbance, and mental health problems [2-4]. These unhealthy habits are associated with a higher risk of noncommunicable diseases (NCDs) [5-7] and greater vulnerability to SARS-CoV-2 infection, resulting in more severe COVID-19 outcomes [8-10].

In Vietnam, the COVID-19 control policies have exerted a considerable impact on people’s lifestyles [11]. The stringent isolation measures have resulted in significant changes in daily routines. For example, symptomatic patients underwent isolation for a minimum of 14 days in designated facilities such as medical camps or field hospitals, where living conditions were limited. This was followed by an additional 14 days of quarantine at home after recovery [12]. These changes are particularly noticeable in alterations to diet and physical activities [13]. Even asymptomatic individuals with COVID-19 (those testing positive for SARS-CoV-2 without symptoms) quarantined for at least 14 days at home are susceptible to a sedentary lifestyle, primarily due to the limited physical activity options and dependence on provided foods [14].

Indeed, several publications have indicated that survivors of COVID-19 experience a wide range of health problems after recovery [15], and these problems are often linked to an individual’s lifestyle and health behaviors [16,17]. Additionally, studies have shown that unhealthy lifestyle behaviors tend to cluster, with individuals who engage in 1 unhealthy behavior being more likely to engage in others [18,19]. For example, the co-occurrence of a sedentary lifestyle with excessive substance use, alcohol consumption, and smoking can lead to worse health conditions [18-20], especially for survivors of COVID-19 who are already vulnerable. While several publications have examined the long-term mental and physical health of COVID-19 infection [21,22], there is limited evidence on the unhealthy lifestyles among survivors of COVID-19, that is, who are more likely to engage in these lifestyles. It would be of interest for policy makers to provide timely and targeted health promotion interventions to survivors of COVID-19 to avoid further complications of long-term COVID-19 health problems.

Several studies have found significant differences in the clustering of unhealthy lifestyle behaviors between sex [18,19,23-25]. For instance, one study in the United States investigated the sex differences in lifestyle behaviors among adults with diabetes between 1999 and 2018. The findings revealed that both male participants and female participants with diabetes displayed unhealthy lifestyle behaviors, but the specific behaviors differed by sex. Male patients with diabetes were more likely to smoke and engage in excessive drinking, whereas their counterparts were more likely to be physically inactive and have poor dietary habits [25]. In another study focusing on Indian adults, researchers explored that the prevalence of multiple unhealthy lifestyle behaviors is significant, with a higher tendency for male adults to exhibit the clustering of multiple factors compared to female ones [26].

In Vietnam, sex differences in lifestyle behaviors arise from societal expectations. Male participants often exhibit a higher tendency to participate in risky behavior influenced by socialization, whereas female participants benefit from their cautiousness and adherence to healthy behaviors and health education [27-29]. For instance, within Vietnamese tradition, smoking and drinking might be perceived as integral to the male gender role, serving to demonstrate masculinity and foster social connections [30]. In contrast, societal expectations generally discourage women from engaging in these behaviors, aligning with gender role norms.

Although previous research has extensively investigated various patterns of lifestyle behaviors, little is known about whether these patterns differ among adult survivors of COVID-19. Additionally, previous studies have examined the impact of sociodemographic characteristics such as race, ethnicity, culture, age, socioeconomic status, employment, and education on unhealthy behaviors [31-33]. However, a sex-specific analysis to understanding and measuring these behaviors is still lacking, except in studies conducted on adolescents [23,34]. Therefore, this study aims to use latent class analysis (LCA) to identify distinct patterns of unhealthy lifestyle behaviors, including cigarette smoking, alcohol consumption, unhealthy diet, physical inactivity, and sedentary behaviors, among survivors of COVID-19 after their recovery, focusing on sex differences. Furthermore, this study seeks to identify the factors associated with “more unhealthy” behaviors in this population.

## Methods

### Study Settings

**Figure 1:**
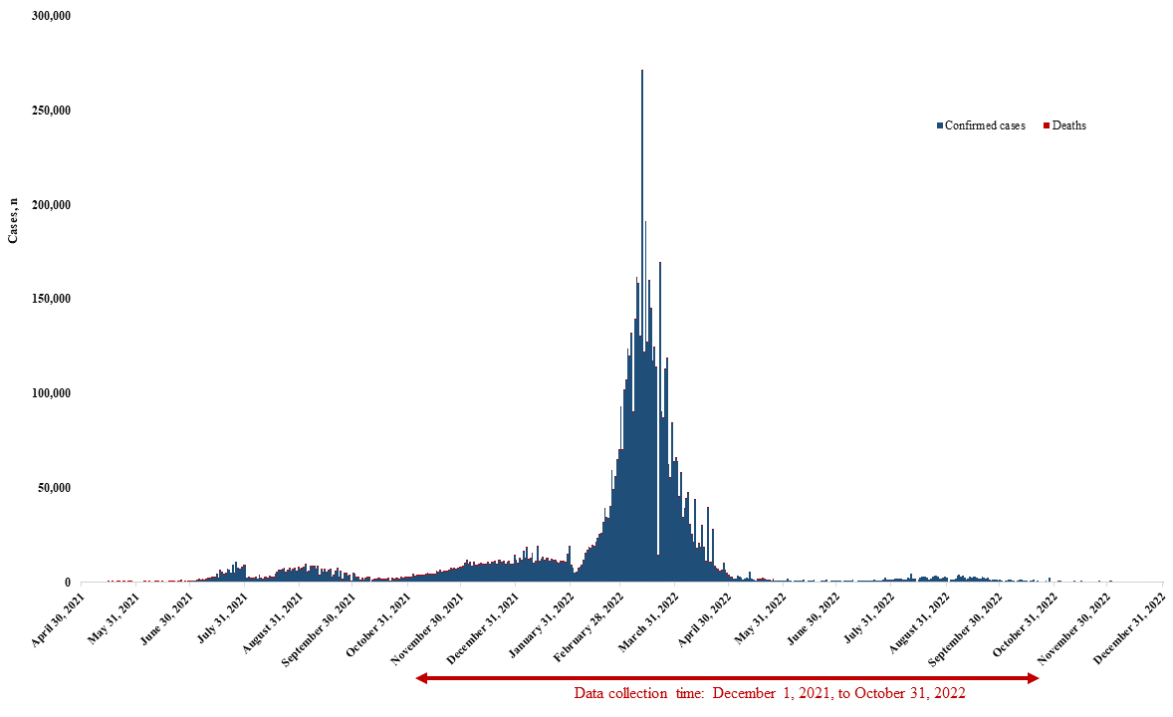
COVID-19 situation in Vietnam from April 2021 to December 2022.

Vietnam first confirmed the presence of the SARS-CoV-2 virus in January 2020, with initial cases being mostly from other countries. However, local transmission began to develop in February and March 2020. To curb the spread, the Vietnamese government implemented a zero–COVID-19 strategy throughout 2020, which involved contact tracing, mass testing, quarantining, and lockdowns. This approach was largely successful, but since April 2021, the country has been facing its largest outbreak yet. As a result, lockdowns have been implemented in one-third of provinces and cities, affecting roughly one-third of the population. The emergence of the Omicron variant in the first quarter of 2022 led to a sharp increase in infections, although Vietnam’s high vaccination rates resulted in fewer fatalities [35] (Figure 1).

Our study collection period spanned from December 1, 2021, to October 31, 2022. The research was carried out across 17 hospitals and health centers located in 9 provinces across the country. We included 5 provinces with 8 hospitals in the Northern region, 3 provinces with 3 health centers in the Central region, and 2 provinces with 6 hospitals and health centers in the Southern region (Multimedia Appendix 1).

### Study Design and Participants

This is a nationwide cross-sectional, web-based survey conducted from 2021 to 2022 in Vietnam. We enrolled Vietnamese adults (aged 18 years or older) who had been infected with SARS-CoV-2, which is confirmed by available positive test results for SARS-CoV-2 (either real-time polymerase chain reaction or rapid antigen test), and afterward recovered from the infection, which is confirmed by available negative test results for SARS-CoV-2 (either real-time polymerase chain reaction or rapid antigen test). Participants who did not consent, were nonpermanent residents of Vietnam, were unable to understand the survey questions, or had cognitive or mental health issues that may have affected their responses to the survey were excluded from the study (n=87).

**Figure 2:**
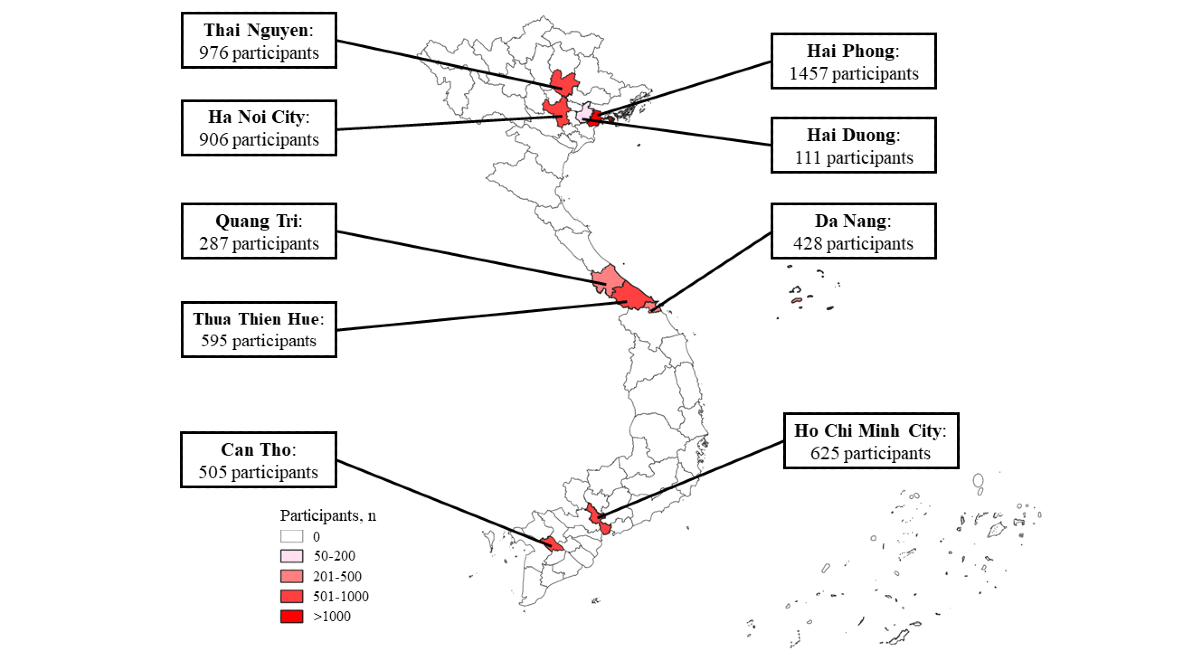
Distribution of participants in the study.

Participants from 17 hospitals and COVID-19 health centers across 9 provinces representative of 3 geographical regions of Vietnam were purposively selected. We recruited 3450 participants in 4 Northern region provinces and cities, 1130 participants in 3 Central region provinces, and 1310 participants in 2 Southern region cities. In total, we collected data from 5890 participants. Details of the number of participants recruited per site of the 3 geographical regions can be found in Figure 2 and Multimedia Appendix 1.

### Sample Size

A sample of 5890 participants provided sufficient data for inclusion in the analysis, representing 50.1% of the total 11,761 survivors of COVID-19 from 17 study sites and 0.05% of the total 10,607,166 survivors of COVID-19 in Vietnam until the end of data collection on October 31, 2022 [35].

### Data Collection Procedures

The data were collected from December 1, 2021, to October 31, 2022. The survey form was prepared on Microsoft Forms (Microsoft Corp). Initially, we secured agreements to collaborate with 17 target sites for the research. Before data collection, senior researchers provided the enumerators, who were health care workers at each site, with an introduction to the data collection procedures. The enumerators reached out to survivors of COVID-19 through various contact channels (phone, smartphone app, and email) and distributed the survey link. The reminders were sent out to achieve the response rate. Data were collected, extracted, and coded for analysis anonymously.

### Assessments and Measurements

#### LCA Indicators: Unhealthy Lifestyle Behaviors

Based on previous literature, we assessed 5 most common unhealthy lifestyle behaviors including the following binary indicators:

- Cigarette smoking: Participants were asked about their tobacco use status and were given 3 options: “currently smoking,” “used to smoke and stopped,” and “never smoke.” We coded the variable “cigarette smoking” as “nonsmokers” if participants answered “never smoke” or “used to smoke and stopped.” Participants who answered “currently smoking” were categorized as “smokers.”
- Alcohol consumption: We asked participants whether they had consumed at least 1 standard drink of alcoholic beverage in the past 30 days. The variable was binary coded as “yes” or “no.”
- Unhealthy diet: We used the 5-item healthy eating score to assess the healthy diet. The 5-item healthy eating score is comparable with the 2015 health eating index in assessing the overall diet quality [36] and has been validated and used in Vietnam [37]. Participants were asked to respond to 5 questions about their consumption frequency of fruits, vegetables, whole grains, dairy, and fish over the past 30 days using a 6-point scale ranging from 0=rarely or never to 5=3 or more times per day. The total score can range from 0 to 25, with a higher score indicating a healthier diet. We used a cut-off point as the median of 15 (IQR 13-17) to create a binary variable, with a score of less than 15 indicating the “unhealthy diet.”
- Physical inactivity: Physical activity is defined as any bodily movement that requires the contraction of skeletal muscles and increases energy expenditure above the resting metabolic rate [38]. In this study, we used the short version of the International Physical Activity Questionnaire to assess participants’ physical activity levels [39]. Participants who reported engaging in vigorous-intensity activity on 3 or more days per week, or moderate-intensity activity or walking for at least 30 minutes per day on 5 or more days, were considered to have met the criteria for being “physically active,” and the opposite was labeled “physically inactive.”
- Sedentary behaviors: Sedentary behaviors are defined as any activities that involve sitting or reclining and consume an energy expenditure equal to or below 1.5 metabolic equivalents. Sitting time is usually the main indicator used to quantify the time devoted to sedentary behaviors [40]. To assess sedentary behavior, we asked survivors of COVID-19 about the average number of hours they spent sitting for nonwork purposes (eg, watching television, playing computer games, using social media, or engaging in other sitting activities) on a typical day in the past 7 days. Participants who reported spending more than 2 hours per day on these activities were classified as having sedentary behavior [34].

#### Clinical Parameters

Participants were asked to report their height in meters and weight in kilograms. The self-reported weight is concordant with image-captured weight in web-based research [41]. The BMI was calculated by dividing their weight by the square of their height in meters. Participants were then classified as underweight (BMI<18.5 kg/m^2^), normal weight (18.5 kg/m^2^≤BMI<23 kg/m^2^), overweight (23 kg/m^2^≤BMI<25 kg/m^2^), or obese (BMI≥25 kg/m^2^) [42]. In addition, comorbid conditions other than COVID-19 were also assessed.

#### Sociodemographic Characteristics

Sociodemographic characteristics were also measured, including age (years), sex (male or female), marital status (single; married; or widow, divorced, or separated), education levels (illiterate or elementary, secondary or high school, vocational or college, or university or higher), employment status (dependent, independent, or unemployed), whether the participant was a health care worker (yes or no), and self-reported social status (low, middle, or high).

### Data Analysis

Data were analyzed on Stata (version 17.0; StataCorp). First, we tabulated sociodemographic characteristics and nutritional status for the overall sample separately by sex and differentiated by Wilcoxon rank-sum tests, Pearson chi-square tests, and Fisher exact tests. Second, the prevalence and 95% CIs of 5 unhealthy lifestyle behaviors were calculated for male and female participants. The 95% CIs were calculated using the formula CI=proportion mean±1.96×SE of proportion mean [43]. We then used the UpSet diagrams to describe the combinations of these behaviors [44]. The UpSet diagrams can help display complex intersections of a lifestyle behaviors matrix, where the rows represent different sets of combinations and the columns represent the number of male and female participants who had these combinations.

LCA is a statistical method used to identify subgroups of individuals based on observed variables [45]. Previous studies have used LCA to identify subgroups with distinct patterns of lifestyle behaviors and explore the factors associated with these patterns [31,34]. We used the expectation-maximization algorithm of LCA with 30 iterations—a general optimization technique for deriving maximum likelihood estimates in the presence of latent variables from 5 indicators [46]. This algorithm alternated between the expectation step—imputing latent variables based on current parameter estimates, and the maximization step—updating parameters to maximize the likelihood of the observed data from these 5 indicators [46,47]. Our approach involved systematically testing a series of models with an increasing number of latent classes to pinpoint the most fitting model. We considered Akaike information criterion (AIC) and Bayesian information criterion (BIC) to choose the optimal number of classes. The class with the smallest AIC and BIC was considered a good fit [33].

We defined 2 distinct classes, namely, the “less unhealthy” group and the “more unhealthy” group. Within each latent group, we assessed the predicted probabilities for categorical indicators and assigned each participant to the group with the highest probability. The distribution of indicators and covariates was compared among each group membership, and logistic regression analysis was performed to estimate the association between covariates and being in the “more unhealthy” group, stratified by sex. The selection of covariates for this model was informed by our hypothesis of correlation, guided by the findings in the literature [4,16,18,19,23,25,29]. Odds ratio (OR) and 95% CIs were reported. Likelihood ratio tests were used to determine the effect of each variable in the models.

### Ethical Considerations

This study was reviewed and approved by the institutional ethical review committee of Hanoi University of Public Health, Vietnam (IRB 400/2021/YTCC-HD3 and 45/2022/YTCC-HD3). Participants have consented their participation. Data were confidentially collected and analyzed. No compensation was provided for participation.

## Results

### Participants’ Characteristics

Table 1 provides a summary of participants’ characteristics, stratified by sex. Among 5890 survivors of COVID-19, the median age was 31 (IQR 23-40) years. More than half of them were female (n=3443, 58.5%), married (n=3402, 57.8%), and had attained a university education or higher (n=3034, 51.5%). In total, 3475 (59%) participants were dependent workers, and 1928 (32.7%) were health care workers. Most participants belonged to the middle social status category (n=4822, 81.9%) and had no comorbidities other than COVID-19 (n=4177, 70.9%).

**Table 1:** Distribution of sociodemographic characteristics among 5890 survivors of COVID-19, stratified by sex.

Characteristics		All participants (N=5890)	Male participants (n=2447, 41.5%)	Female participants (n=3443, 58.5%)	*P* value^a^	
Age (years), median (IQR)		31 (23-40)	30 (23-40)	32 (24-41)	<.001	
**Sex, n (%)**						
	Male	2447 (41.5)	N/A^b^	N/A	N/A	
	Female	3443 (58.5)	N/A	N/A	N/A	
**Marital status, n (%)**						<.001
	Single	2346 (39.8)	1156 (47.2)	1190 (34.6)		
	Married	3402 (57.8)	1259 (51.5)	2143 (62.2)		
	Widow or divorce or separate	142 (2.4)	32 (1.3)	110 (3.2)		
**Education levels, n (%)**						<.001
	Illiterate or elementary	161 (2.7)	62 (2.5)	99 (2.9)		
	Secondary or high school	1264 (21.5)	569 (23.3)	695 (20.2)		
	Vocational or college	1431 (24.3)	425 (17.4)	1006 (29.2)		
	University or higher	3034 (51.5)	1391 (56.8)	1643 (47.7)		
**Employment status, n (%)**						.003
	Dependent worker	3475 (59)	1382 (56.5)	2093 (60.8)		
	Independent worker	1461 (24.8)	656 (26.8)	805 (23.4)		
	Unemployed	954 (16.2)	409 (16.7)	545 (15.8)		
**Health care workers, n (%)**						<.001
	No	3962 (67.3)	1790 (73.2)	2172 (63.1)		
	Yes	1928 (32.7)	657 (26.8)	1271 (36.9)		
**Social status, n (%)**						<.001
	Low	767 (13)	302 (12.3)	465 (13.5)		
	Middle	4822 (81.9)	1974 (80.7)	2848 (82.7)		
	High	301 (5.1)	171 (7)	130 (3.8)		
**Comorbidity, n (%)**						.09
	No	4177 (70.9)	1764 (72.1)	2413 (70.1)		
	Yes	1713 (29.1)	683 (27.9)	1030 (29.9)		
**BMI (kg/m^2^), n (%)**						<.001
	Underweight	709 (12)	133 (5.4)	576 (16.7)		
	Normal	3573 (60.7)	1261 (51.5)	2312 (67.2)		
	Overweight	1043 (17.7)	671 (27.4)	372 (10.8)		
	Obese	565 (9.6)	382 (15.6)	183 (5.3)		

^a^*P* value from Wilcoxon rank-sum, Pearson chi-square, and Fisher exact test compared the distribution of covariates between male and female participants.

^b^N/A: not applicable.

There were significant differences in the distribution of all covariates between male and female participants except for comorbidities. A higher percentage of female participants were underweight (female: 576/3443, 16.7% vs male: 133/2447, 5.4%). In contrast, male participants had higher rates of overweight (male: 671/2447, 27.4% vs female: 372/3443, 10.8%) and obesity (male: 382/2447, 15.6% vs female: 183/3443, 5.3%) than female participants.

### Distribution of Unhealthy Lifestyle Behaviors

Table 2 displays the proportion of unhealthy lifestyle behaviors in survivors of COVID-19, stratified by sex. Among both sexes, sedentary behaviors were the most prevalent behavior (male: 1871/2447, 76.5% and female: 2629/3443, 76.4%), followed by unhealthy diet (male: 1508/2447, 61.6% and female: 2109/3443, 61.3%). The prevalence of smoking and drinking was significantly higher among male participants compared to their female counterparts (307/2447, 12.5% vs 12/3443, 0.3%; *P*<.001 and 1217/2447, 49.7% vs 816/3443, 23.7%; *P*<.001, respectively). Conversely, the proportion of physical inactivity among female participants was higher than among male participants (female: 1928/3443, 56% vs male: 988/2447, 40.4%; *P*<.001).

**Table 2:** Distribution of 5 unhealthy lifestyle behaviors, stratified by sex (N=5890).

Unhealthy lifestyle behaviors		All participants (N=5890), n (%)	Male participants (n=2447, 41.5%), n (%)	Female participants (n=3443, 58.5%), n (%)	*P* value^a^	
**Cigarette smoking**						<.001
	Nonsmokers	5571 (94.6)	2140 (87.5)	3431 (99.7)		
	Smokers	319 (5.4)	307 (12.5)	12 (0.3)		
**Alcohol consumption**						<.001
	No	3857 (65.5)	1230 (50.3)	2627 (76.3)		
	Yes	2033 (34.5)	1217 (49.7)	816 (23.7)		
**Unhealthy diet**						.77
	No	2273 (38.6)	939 (38.4)	1334 (38.7)		
	Yes	3617 (61.4)	1508 (61.6)	2109 (61.3)		
**Physical inactivity**						<.001
	No	2974 (50.5)	1459 (59.6)	1515 (44)		
	Yes	2916 (49.5)	988 (40.4)	1928 (56)		
**Sedentary behavior**						.93
	No	1390 (23.6)	576 (23.5)	814 (23.6)		
	Yes	4500 (76.4)	1871 (76.5)	2629 (76.4)		

^a^*P* value from Pearson chi-square and Fisher exact test compared the distribution of covariates between male and female participants.

Table 3 illustrates the distribution of co-occurring unhealthy lifestyle behaviors among male and female participants. Almost all survivors of COVID-19 had participated in 1 behavior (male: 2432/2447, 99.4% and female: 3411/3443, 99.1%). In all categories, there was a higher prevalence of male participants than female participants who had 2 (male: 1918/2447, 78.4% vs female: 2551/3443, 74.1%), 3 (male: 1091/2447, 44.6% vs female: 1243/3443, 36.1%), 4 (male: 331/2447, 13.5% vs female: 282/3443, 8.2%), and all 5 (male 61/2447, 2.5% vs female: 3/3443, 0.1%) behaviors. The absolute and relative differences between the prevalence of male and female participants also increased as the number of behaviors increased. The ratio of male to female participants participating in 1 behavior was 1.003. This ratio increased gradually with a higher number of behaviors and surged to 25 at 5 unhealthy behaviors.

**Figure 3:**
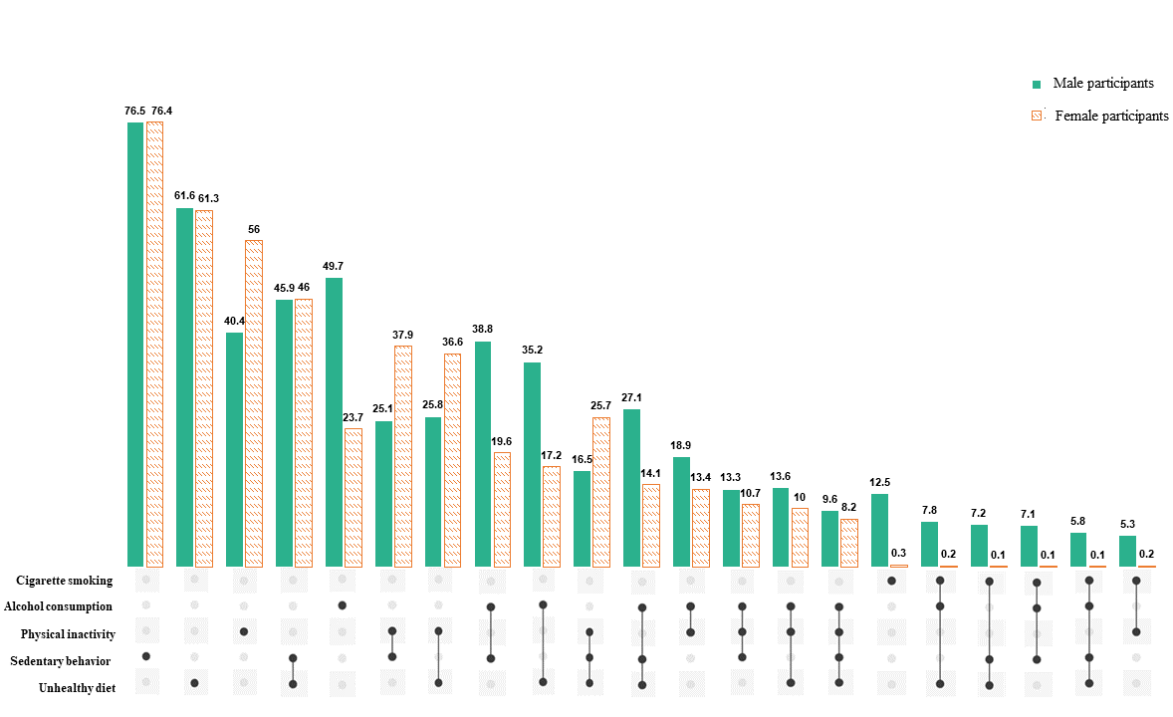
UpSet diagrams for different combinations of unhealthy lifestyle behaviors of participants, stratified by sex. This figure does not illustrate the combination with a prevalence of less than 5% in both sexes. The table underneath depicts the co-occurrence of unhealthy lifestyle behavior combination, where the rows represent different sets of combinations and the columns represent the number of male and female participants who had these combinations.

Figure 3 illustrates different combinations of unhealthy behaviors, stratified by sex. The most frequent combination observed in 1123 (45.9%) of 2447 male participants and 1584 (46%) of 3443 female participants was sedentary behavior and unhealthy diet. Male participants showed a high prevalence of combining alcohol consumption with sedentary behaviors (949/2447, 38.8%) and an unhealthy diet (861/2447, 35.2%), followed by the combination of all 3 abovementioned behaviors (663/2447, 27.1%). On the other hand, female participants tended to combine physical inactivity with sedentary behavior (1305/3443, 37.9%) or an unhealthy diet (1260/3443, 36.6%), as well as all 3 behaviors (885/3443, 25.7%).

**Table 3:** Distribution of unhealthy lifestyle behavior co-occurrence, stratified by sex (N=5890).

Unhealthy lifestyle behavior co-occurrence		All participants (N=5890), n (%)	Male participants (n=2447, 41.5%), n (%)	Female participants (n=3443, 58.5%), n (%)	% Difference (absolute; relative)		*P* value^a^	
**One risk**						0.3 (1.003)		.18
	No	47 (0.8)	15 (0.6)	32 (0.9)				
	Yes	5843 (99.2)	2432 (99.4)	3411 (99.1)				
**Two risks**						4.3 (1.06)		<.001
	No	1421 (24.1)	529 (21.6)	892 (25.9)				
	Yes	4469 (75.9)	1918 (78.4)	2551 (74.1)				
**Three risks**						8.5 (1.24)		<.001
	No	3556 (60.4)	1356 (55.4)	2200 (63.9)				
	Yes	2334 (39.6)	1091 (44.6)	1243 (36.1)				
**Four risks**						5.3 (1.65)		<.001
	No	5277 (89.6)	2116 (86.5)	3161 (91.8)				
	Yes	613 (10.4)	331 (13.5)	282 (8.2)				
**Five risks**						2.4 (25.0)		<.001
	No	5826 (98.9)	2386 (97.5)	3440 (99.9)				
	Yes	64 (1.1)	61 (2.5)	3 (0.1)				

^a^*P* value from Pearson chi-square and Fisher exact test compared the distribution of covariates between male and female participants.

### The Cluster of Unhealthy Lifestyle Behaviors

Table 4 reports the model fit indices of LCA models from 2 to 6 classes fitting to 5 behavior indicators. The 2-class model has the lowest AIC and BIC. In addition, the 2-class model is easier to interpret than the other models. Therefore, we proceed with the 2-class model.

**Table 4:** The model fit indices of latent class analysis models.

Variable	Two-class model	Three-class model	Four-class model	Five-class model	Six-class model
Log likelihood	–16,050.62	–15,787.33	–15,769.49	–15,768.81	Not convergent
AIC^a^	31,584.99	31,608.67	32,123.24	31,595.62	Not convergent
BIC^b^	31,722.26	32,196.75	31,738.67	31,789.39	Not convergent

^a^AIC: Akaike information criterion.

^b^BIC: Bayesian information criterion.

Table 5 presents the 2-class membership, distributed among the prevalence of 5 unhealthy lifestyle behaviors and stratified by sex. The 2 classes were labeled based on the distribution of each behavior in each class. The “less unhealthy” group included 64.7% (3810/5890) of total participants, and among them, there were 48.4% (1184/2447) of male participants and 76.3% (2626/3443) of female participants. Sedentary behavior was the most common behavior exhibited in both male and female participants in this group (887/1184, 74.9% and 1954/2626, 74.4%, respectively), followed by an unhealthy diet and physical inactivity. None of the participants in this membership had participated in 4 or 5 behaviors, and none of them reported any alcohol consumption. More than 43% of both male (521/1184, 43.8%) and female (1143/2626, 43.5%) “less unhealthy” participants engaged in 2 behaviors.

**Table 5:** Distribution of unhealthy lifestyle behaviors among survivors of COVID-19 by 2-class memberships.

Unhealthy lifestyle behaviors		All participants (N=5890)			Male participants (n=2447)			Female participants (n=3443)	
		Less unhealthy (n=3810, 64.7%), n (%)	More unhealthy (n=2080, 35.3%), n (%)	Less unhealthy (n=1184, 48.4%), n (%)		More unhealthy (n=1263, 51.6%), n (%)	Less unhealthy (n=2626, 76.3%), n (%)		More unhealthy (n=819, 23.8%), n (%)
**Unhealthy lifestyle behaviors**									
	Cigarette smoking	22 (0.6)	297 (14.3)	19 (1.6)		288 (22.8)	3 (0.1)		9 (1.1)
	Alcohol consumption	0 (0)	2036 (97.7)	0 (0)		1217 (96.4)	0 (0)		816 (99.9)
	Unhealthy diet	2119 (55.6)	1498 (72)	602 (50.7)		908 (71.9)	1519 (57.8)		590 (72.2)
	Physical inactivity	1977 (51.9)	939 (45.1)	509 (43)		497 (37.9)	1468 (55.9)		460 (56.3)
	Sedentary behaviors	2841 (74.6)	1659 (79.8)	887 (74.9)		984 (77.9)	1954 (74.4)		675 (82.6)
**Number of unhealthy lifestyle behaviors**									
	0	47 (1.2)	0 (0)	15 (1.3)		0 (0)	32 (1.2)		0 (0)
	1	1338 (35.1)	36 (1.7)	489 (41.3)		25 (2)	849 (32.3)		11 (1.3)
	2	1662 (43.6)	473 (22.7)	521 (43.8)		308 (24.4)	1143 (43.5)		165 (20.2)
	3	763 (20)	958 (46.1)	161 (13.6)		599 (47.4)	602 (22.9)		359 (43.9)
	4	0 (0)	549 (26.4)	0 (0)		270 (21.4)	0 (0)		279 (34.1)
	5	0 (0)	64 (3.1)	0 (0)		61 (4.8)	0 (0)		3 (0.4)

The “more unhealthy” group included 35.3% (2080/5890) of total participants, and among them, there were 51.6% (1263/2447) of male participants and 23.8% (819/3443) of female participants. The most prevalent behavior practiced in both male and female participants was alcohol consumption (1217/1263, 96.4% and 816/819, 99.9%, respectively), followed by sedentary behaviors (984/1263, 77.9% and 675/819, 82.6%, respectively). Male participants in the “more unhealthy” group exhibited a significantly higher prevalence of cigarette smoking compared to their female counterparts (288/1263, 22.8% vs 9/819, 1.1%, respectively). There were 599 (47.4%) of 1263 male participants and 359 (43.9%) of 819 female participants in this group who participated in 3 unhealthy behaviors. Approximately 5% (61/1263) of male participants engaged in all 5 behaviors, while this proportion was 0.4% (3/819) among female participants in the same class.

Table 6 displays the distribution of covariates by class membership. There were significant differences in the distribution of all covariates between “less unhealthy” and “more unhealthy” groups. Nearly half (1010/2080, 48.6%) of the participants in the “more unhealthy” group were single, 60.7% (1263/2080) of them were male, 67.3% (1400/2080) of them had at least a university education, and 71.9% (1495/2080) of “more unhealthy” participants were dependent workers. Regarding “less unhealthy” group, nearly 69% (2626/3810) of the participants were female, and 62.4% (2377/3810) were married. More than half (1980/3810, 52%) of “less unhealthy” participants were dependent workers, and 42.9% (1634/3810) of them had at least a university education. The prevalence of obesity was almost 3 times higher in the “more unhealthy” group compared to the “less unhealthy” group (314/2080, 15.1% vs 251/3810, 6.6%, respectively).

**Table 6:** Distribution of covariates by class membership (N=5890).

Covariates		Less unhealthy group (n=3810, 64.7%)	More unhealthy group (n=2080, 35.3%)	*P* value^a^	
Age (years), median (IQR)		32.0 (23.0-44.0)	29.0 (23.0-37.0)	<.001	
**Sex, n (%)**					<.001
	Male	1184 (31.1)	1263 (60.7)		
	Female	2626 (68.9)	817 (39.3)		
**Marital status, n (%)**					<.001
	Single	1336 (35.1)	1010 (48.6)		
	Married	2377 (62.4)	1025 (49.3)		
	Widow or divorce or separate	97 (2.5)	45 (2.2)		
**Education levels, n (%)**					<.001
	Illiterate or elementary	140 (3.7)	21 (1)		
	Secondary or high school	1025 (26.9)	239 (11.5)		
	Vocational or college	1011 (26.5)	420 (20.2)		
	University or above	1634 (42.9)	1400 (67.3)		
**Employment status, n (%)**					<.001
	Dependent worker	1980 (52)	1495 (71.9)		
	Independent worker	1139 (29.9)	322 (15.5)		
	Unemployed	691 (18.1)	263 (12.6)		
**Health care workers, n (%)**					<.001
	No	2699 (70.8)	1263 (60.7)		
	Yes	1111 (29.2)	817 (39.3)		
**Social status, n (%)**					<.001
	Low	529 (13.9)	238 (11.4)		
	Middle	3115 (81.8)	1707 (82.1)		
	High	166 (4.4)	135 (6.5)		
**Comorbidity, n (%)**					.004
	No	2654 (69.7)	1523 (73.2)		
	Yes	1156 (30.3)	557 (26.8)		
**Nutritional status, n (%)**					<.001
	Underweight	495 (13)	214 (10.3)		
	Normal	2453 (64.4)	1120 (53.8)		
	Overweight	611 (16)	432 (20.8)		
	Obese	251 (6.6)	314 (15.1)		

^a^*P* value from Pearson chi-square and Fisher exact test.

### Factors Related to the More Unhealthy Group

Table 7 presents multivariable logistics regression to examine the association between covariates and being in the “more unhealthy” group among male and female participants. In both sexes, younger individuals had lower odds of being in the “more unhealthy” group (male: *P*=.001; OR 0.99, 95% CI 0.98-0.99 and female: *P*<.001; OR 0.98, 95% CI 0.97-0.99). Marital status showed a significant association with the “more unhealthy” group in both models. Married male participants had higher odds of being in the “more unhealthy” group compared to single ones (OR 1.45, 95% CI 1.14-1.85), while female participants exhibited lower odds (OR 0.65, 95% CI 0.51-0.83). Independent workers and unemployed participants had lower odds of being in the “more unhealthy” group compared to dependent workers of both sexes. In both models, higher educational levels were found to be associated with increased odds of belonging to the “more unhealthy” group.

**Table 7:** Multivariable models of factors related to more unhealthy group among survivors of COVID-19.

Factors		Male participants			Female participants	
		OR^a^ (95% CI)	*P* value^a^	OR (95% CI)		*P* value^b^
Age (years)		0.99 (0.98-0.99)	.001	0.98 (0.97-0.99)		<.001
**Marital status (reference: single)**			.006			<.001
	Married	1.45 (1.14-1.85)		0.65 (0.51-0.83)		
	Widow or divorce or separate	2.11 (0.94-4.75)		1.16 (0.68-1.98)		
**Education levels (reference: illiterate or elementary)**			<.001			<.001
	Secondary or high school	1.05 (0.59-1.88)		3.06 (0.72-12.9)		
	Vocational or college	1.92 (1.06-3.47)		4.64 (1.11-19.39)		
	University or above	2.4 (1.34-4.31)		8.23 (1.97-34.38)		
**Current employment status (reference: dependent workers)**			<.001			<.001
	Independent workers	0.65 (0.51-0.82)		0.37 (0.27-0.51)		
	Unemployment	0.55 (0.42-0.72)		0.44 (0.32-0.59)		
**Being health care workers (reference: no)**			.52			.36
	Yes	1.08 (0.86-1.36)		0.91 (0.74-1.12)		
**Social status (reference: low)**			.96			.66
	Middle	1.03 (0.80-1.33)		1.13 (0.87-1.47)		
	High	1.06 (0.71-1.58)		1.09 (0.68-1.76)		
**Comorbidity (reference: no)**			.06			.98
	Yes	1.21 (0.99-1.47)		1.00 (0.83-1.22)		
**Nutritional status (reference: normal)**			<.001			.005
	Underweight	0.6 (0.41-0.89)		1.11 (0.89-1.39)		
	Overweight	1.04 (0.85-1.26)		1.42 (1.07-1.87)		
	Obese	2.03 (1.58-2.62)		1.73 (1.21-2.47)		

^a^OR: odds ratio.

^b^*P* value from likelihood ratio test.

Furthermore, individuals who are overweight or obese were more likely to fall into the “more unhealthy” category when compared to participants with a normal weight. Among male participants, the odds of being in the “more unhealthy” group slightly increased for overweight individuals who are overweight (OR 1.04, 95% CI 0.85-1.26), while significantly higher odds were found for individuals who are obese (OR 2.03, 95% CI 1.58-2.62). For female participants, both individuals who are overweight (OR 1.42, 95% CI 1.07-1.87) and obese (OR 1.73, 95% CI 1.21-2.47) had an increased likelihood of being in the “more unhealthy” group. Female participants who are underweight showed a higher likelihood of falling into the “more unhealthy” group (OR 1.11, 95% CI 0.89-1.39), but this was not the case for male participants (OR 0.6, 95% CI 0.41-0.89). Being a health care worker, level of social status, and having comorbidities did not show a statistically significant association with higher odds of being in the “more unhealthy” group.

## Discussion

### Principal Findings

This national study examines the prevalence and clustering patterns of 5 unhealthy lifestyle behaviors among survivors of COVID-19 in Vietnam. This study provides a distinct pattern of unhealthy behaviors exhibited by male and female individuals after their COVID-19 recovery. We found that sedentary behaviors and unhealthy diets are common unhealthy behaviors in both sexes. While physical inactivity was more commonly practiced among female participants, male participants tended to engage in smoking and alcohol consumption. Nearly all participants had engaged in at least 1 unhealthy behavior, and male participants had a higher likelihood of engaging in multiple behaviors than female participants. Male participants also tended to practice alcohol intake with others such as sedentary behaviors and unhealthy diet, whereas female participants engaged in physical inactivity with sedentary behaviors and unhealthy diet.

Two unhealthy behavior classes were identified using LCA, with the “more unhealthy” group featured by a greater proportion of male participants. Certain common factors associated with this group were observed in both male and female participants, including older age, dependent employment, high educational levels, and obesity. However, sex-specific differences were found. For male participants, being married was an additional factor linked to a higher likelihood of being in the “more unhealthy” group. On the other hand, for female participants, being single and underweight were additional factors associated with this group.

### Prevalence of Unhealthy Lifestyle Behaviors by Sex

The most prevalent unhealthy behaviors among survivors of COVID-19 were sedentary behaviors and unhealthy diets, which is consistent with findings from other studies among students and younger adults [23,34,48]. While there were significant differences between the percentage of male and female participants engaged in almost all behaviors, there were no significant differences between the prevalence of unhealthy diet and sedentary behavior in male and female participants in our sample. Our findings are in line with those from a study conducted among 1058 first-year students in Greece in 2016, which also found no significant differences in the prevalence of sedentary behavior (defined by screen time of more than 2 hours) and fruit and vegetable intake between male and female participants [23]. Since the study collected data when several provinces and cities were under lockdown across Vietnam, one explanation for the absence of these significant sex differences in terms of sedentary behavior and an unhealthy diet could be that both sexes were impacted similarly in this period. The lockdown might have caused significant changes in daily routines and lifestyles for the whole population, such as reduced physical activity, increased sedentary behavior, and changes in dietary patterns. Similar changes were observed in previous studies that took place during the cordon sanitaire period globally [49-51]. These changes can then exacerbate the prevalence of both sexes engaging in unhealthy behaviors. Indeed, there was a considerable increase in snacking and meal numbers or in unfavorable food choices and dietary habits during the COVID-19 lockdown reported in 2020, and there were no significant differences in sex participation rate as well [52].

Our evidence highlights the significant sex disparities in cigarette smoking and alcohol consumption among survivors of COVID-19, skewing higher in male participants. This observation aligns with prior research, where male participants tend to engage in more hazardous behaviors than female participants [53-56]. Male participants were also more likely to combine alcohol consumption with other factors such as sedentary lifestyles and unhealthy diets. With nearly 30% (683/2447) of male participants in our sample with chronic conditions, the high prevalence of co-occurring unhealthy behaviors coupled with the possible long-term effect of COVID-19 infection might increase the risk of NCDs in this population [57]. The observed sex differences in cigarette smoking and alcohol consumption in this study might be explained by a range of social, cultural, and economic factors. Vietnamese tradition perceived smoking and drinking as a part of the male gender role, used to display masculinity and establish social connections [30], while women are generally not encouraged to engage in these behaviors due to gender role expectations. Following such a drastic health event such as a cancer diagnosis or in our case—COVID-19 infection, lifestyle behaviors might also differ between sexes [58]. While women are more likely to adhere to health education and healthy behaviors after diagnosis, men are less likely to change their behaviors [28]. In addition, possible stress and anxiety induced by both the infection and the pandemic might cause men to smoke or drink more as a coping mechanism [59]. To address this gap, targeted interventions for male survivors of COVID-19 are recommended. These interventions may include a health education program for health problems following COVID-19 infection focused on the consequences associated with smoking and alcohol intake or a health promotion campaign highlighting the double-burden aspects of long COVID-19 symptoms and unhealthy behaviors.

### Clustering of Multiple Unhealthy Lifestyle Behaviors by Sex

We highlighted significant differences in the number and combination of lifestyle-compromising behaviors between male and female participants. Surprisingly, the prevalence of male participants engaging in 4 or 5 unhealthy lifestyle behaviors simultaneously was nearly twice and 25 times higher compared to female participants. These findings are consistent with evidence from the adult population in India, which suggests that more adult male participants tend to exhibit the clustering of multiple NCD risk factors than female participants [26]. However, these findings contradict previous research indicating that female participants tend to engage in multiple unhealthy behaviors more frequently than male participants [23,34]. For example, in a study involving 3495 adolescents in Vietnam, it was found that the prevalence of practicing 2 unhealthy behaviors was similar between male and female participants. However, there was a sex discrepancy in the co-occurrence of 3 factors (male: 27.3% and female: 31.5%) and 4 factors (male: 13.7% and female: 15.1%) [34]. Similarly, a study conducted with 1058 first-year students in Greece found that the co-occurrence of 4 unhealthy factors was twice as high in female participants compared to male participants (male: 2.9% and female: 5.2%) [23]. It is important to note that the percentages of co-occurring behaviors may vary across studies due to differences in the number and types of behaviors measured as well as the specific cut-off points used. This is particularly relevant in cases where clear guidelines are lacking [32,60].

Among survivors of COVID-19 in Vietnam, we identified 2 distinct behavior patterns: “less unhealthy” and “more unhealthy” groups. These groups differed significantly in various factors. In the “less unhealthy” group, which represented around 65% (3810/5890) of the sample, a sedentary lifestyle was most prevalent, and there were a higher proportion of female participants. Conversely, the “more unhealthy” group, constituting over one-third of the population, was predominantly male and exhibited higher rates of alcohol intake and sedentary behavior. Additionally, the 2 groups differed in the number of concurrent unhealthy lifestyle behaviors. None of the “less unhealthy” participants reported engaging in more than 3 unhealthy behaviors simultaneously, while over 70% (male: 930/1263, 73.6% and female: 642/819, 78.4%) of the “more unhealthy” ones reported 3 or more behaviors. These distinctions were also reflected in the sex distribution. This finding is in line with a previous study documenting the clustering of health-related behaviors among Indian adults and US young adults [26], in which the authors also found a higher percentage of men grouped in the higher-risk cluster. Overall, the findings reaffirm that (1) there were distinct patterns of unhealthy lifestyle behaviors practiced by survivors of COVID-19, and (2) these patterns differ significantly by sex.

Our findings also indicate that several factors were associated with the “more unhealthy” pattern with sex-specific differences. In this study, male participants who were married displayed a higher likelihood of engaging in unhealthy behaviors compared to those who were single. This contradicts previous research conducted on healthy behaviors among young Korean adults [61] and survivors of cancer [62], which found that unmarried men or men without a partner were more likely to practice unhealthy behaviors compared to their married counterparts. Our evidence suggests that dependent workers, especially male participants, were more likely to engage in unhealthy behaviors. This aligns with previous research indicating that men in dependent jobs, particularly in Asian contexts, tend to participate in social gatherings at work that often involve smoking and drinking [63]. The drinking culture after work is a common practice in many Asian countries, including Vietnam [64], South Korea [65,66], and Japan [67]. On the other hand, self-employment was positively associated with healthier behaviors. This can be attributed to the flexibility self-employed individuals have in managing their work schedule, allowing them to allocate time for health promotion activities [68,69].

Additionally, we found that obesity was significantly associated with the “more unhealthy” group. This highlights the need to address the growing public health challenge of a higher prevalence of obese individuals, which has been exacerbated by the COVID-19 pandemic [70-73]. Given that our adults recovering from COVID-19 are already at risk of postrecovery complications, the engagement in unhealthy behaviors due to obesity conditions further places them in a vulnerable position. We strongly recommend targeted public health interventions and services for this population, focusing on promoting an active lifestyle and a balanced diet and discouraging unhealthy behaviors to regain and maintain their pre-COVID-19 infection health status.

### Limitations and Strengths

This study has several limitations that should be acknowledged. First, the reliance on self-reported data for assessing lifestyle behaviors introduces the possibility of information bias. Participants may have underreported or overreported their engagement in certain behaviors. Second, the cross-sectional design of this study restricts our ability to establish causal relationships between lifestyle behaviors and different demographic factors. Third, the use of web-based surveys may have introduced selection bias, as only individuals who were contactable by phone were included in the sample, and the overall response rate was 50.1% (5890/11,761). It is important to consider the generalizability of our findings, as they may not apply to other vulnerable populations experiencing different chronic health impacts, given the variations in definitions, methodologies, and targeted populations in measuring lifestyle behaviors [32,60]. Fourth, the changes in lifestyle behaviors before and amid the pandemic were not investigated in this study. Fifth, we were unable to access data on Vietnamese societal and cultural dynamics to provide implications for the observed sex differences in lifestyle behaviors. Future research should address these limitations by using other empirical measures of unhealthy lifestyle behaviors and using longitudinal study designs to provide more robust evidence.

Despite certain limitations, this study possesses notable strengths. First, the data were derived from a large population-based cohort, enhancing the representativeness of our findings. Additionally, we used validated questionnaires to assess unhealthy lifestyle behaviors and associated factors, bolstering the generalizability of our study and facilitating comparisons with prior research. Moreover, this study contributes to the existing body of knowledge by shedding light on the distinct patterns of lifestyle behaviors observed among adults recovering from COVID-19, including sex differences, through the advanced application of LCA [55]. As survivors of COVID-19 constitute a growing population facing various health challenges, this study provides valuable insights into their sex-specific engagement in unhealthy behaviors. This, in turn, highlights the importance for public health agencies and policy makers to recognize the inadvertent detrimental impact of the COVID-19 pandemic, as well as the infection itself, on individuals’ lifestyle behaviors and overall health outcomes.

### Conclusions

The study revealed distinct patterns of unhealthy lifestyle behaviors among survivors of COVID-19 in Vietnam, with sedentary lifestyles and unhealthy diets being the most prevalent. Notably, these patterns differed significantly between sexes, with male participants exhibiting higher unhealthy behaviors and engaging in a greater number of unhealthy behaviors compared to female counterparts. These findings have important implications for public health initiatives, highlighting the need for tailored educational interventions that address sex-specific lifestyle behaviors. Specifically, efforts should be made to reduce unhealthy tendencies, particularly among married male adults recovering from COVID-19 who are obese, to promote a healthy and active life after COVID-19.

## Appendix

Multimedia Appendix 1 Participant distribution across study sites.

## Abbreviations

AIC: Akaike information criterion
BIC: Bayesian information criterion
LCA: latent class analysis
NCD: noncommunicable disease
OR: odds ratio
