# Supplementary material for: Sex Differences in Clustering Unhealthy Lifestyles Among Survivors of COVID-19: Latent Class Analysis
Source: JMIR Public Health Surveill. 2024 Apr 2;10:e50189. doi: 10.2196/50189 (PMC10989720; doi:10.2196/50189)
Supplement: Multimedia Appendix 1 [file publichealth_v10i1e50189_app1.docx]

Multimedia Appendix 1: Participant Distribution Across Study Sites.

| **No.** | **Region** | **City/ Province** | **Site** | **No. Participant** |
| --- | --- | --- | --- | --- |
| 1 | Northern | Hai Duong province | Hospital for Tropical Diseases | 111 |
| 2 | Northern | Hai Phong city | Viet Tiep Friendship Hospital | 114 |
|  |  |  | Kien An Hospital | 492 |
|  |  |  | Kien Thuy District Hospital | 497 |
|  |  |  | Hai Phong University of Medicine and Pharmacy Hospital | 354 |
| 3 | Northern | Ha Noi city | Military Hospital 103 | 906 |
| 4 | Northern | Thai Nguyen province | Thai Nguyen National Hospital | 461 |
|  |  |  | Gang Thep Hospital of Thai Nguyen | 515 |
| 5 | Central | Thua Thien Hue city | Thua Thien Hue Centralized Isolation Facilities (T2F0, T3F0) | 595 |
| 6 | Central | Quang Tri province | Trieu Phong District Health Center | 287 |
| 7 | Central | Da Nang city | Da Nang Center for Disease Control and Prevention | 428 |
| 8 | Southern | Ho Chi Minh city | Thu Duc City Health Center | 111 |
|  |  |  | Military Hospital 175, Ministry of National Defense | 514 |
| 9 | Southern | Can Tho city | Binh Thuy Field Hospital | 73 |
|  |  |  | Tuberculosis and Lung Diseases Can Tho Hospital | 200 |
|  |  |  | Field Hospital No.1 | 100 |
|  |  |  | Field Hospital No. 2 | 132 |
